# Planar Metasurfaces Enable High‐Efficiency Colored Perovskite Solar Cells

**DOI:** 10.1002/advs.201800836

**Published:** 2018-08-26

**Authors:** Dong Liu, Lin Wang, Qingyu Cui, L. Jay Guo

**Affiliations:** ^1^ MIIT Key Laboratory of Thermal Control of Electronic Equipment School of Energy and Power Engineering Nanjing University of Science and Technology Nanjing 210094 China; ^2^ Department of Electrical Engineering and Computer Science University of Michigan Ann Arbor MI 48109 USA

**Keywords:** metasurfaces, nanometer thick films, perovskite solar cells, simple planar structure

## Abstract

The achievement of perfect light absorption in ultrathin semiconductor materials is not only a long‐standing goal, but also a critical challenge for solar energy applications, and thus requires a redesigned strategy. Here, a general strategy is demonstrated both theoretically and experimentally to create a planar metasurface absorber comprising a 1D ultrathin planar semiconductor film (replacing the 2D array of subwavelength elements in classical metasurfaces), a transparent spacer, and a metallic back reflector. Guided by derived formulisms, a new type of macroscopic planar metasurface absorber is experimentally demonstrated with light near‐perfectly and exclusively absorbed by the ultrathin semiconductor film. To demonstrate the power and simplicity of this strategy, a prototype of a planar metasurface solar cell is experimentally demonstrated. Furthermore, the device model predicts that a colored planar metasurface perovskite solar cell can maintain 75% of the efficiency of its black counterpart despite the use of a perovskite film that is one order of magnitude thinner. The displayed cell colors have high purities comparable to those of state‐of‐the‐art color filters, and are insensitive to viewing angles up to 60°. The general theoretical framework in conjunction with experimental demonstrations lays the foundation for designing miniaturized, planar, and multifunctional solar cells and optoelectronic devices.

## 1 Introduction

Ultrathin semiconductor materials are promising building blocks for solar photovoltaic (PV)^1^ and photoelectrochemical (PEC)^2^ cells and other solar energy harvesting devices, because they have reduced bulk recombination, higher internal quantum efficiency,^3^, ^4^ and exhibit new properties such as flexibility,^5^, ^6^ compared to their traditional bulk counterparts. Thus, they can increase device performance and reduce material costs, and also have potential for portable and flexible devices. However, the achievement of strong light absorption in such ultrathin materials has been a long‐standing but critical challenge and received extensive research efforts. Studies have sought to use metamaterials^7^, ^8^ where subwavelength building blocks are artificially arranged in 3D configurations, and metasurfaces^9^, ^10^, ^11^ that consist of subwavelength resonators arranged at the 2D interface, a thin transparent dielectric spacer, and a metallic back reflector, to enhance optical absorption. Nevertheless, both metamaterial and metasurface optical absorbers require complex nanopatterning steps that are difficult to scale to large areas. In addition, nanostructured building blocks of these absorbers typically introduce nonradiative recombination losses in PV and PEC cells;^2^, ^4^ and thus, for example, nanowire‐based solar cells usually have lower efficiencies than planar cells made from the same materials.^12^ Therefore, enhancing optical absorption in ultrathin semiconductor materials requires redesigning the strategy.

**Figure 1:**
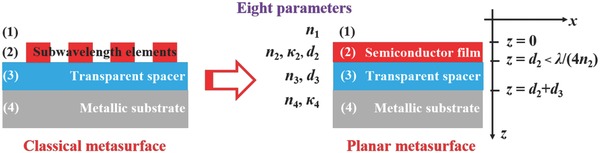
Schematic of a planar metasurface absorber. The absorber consists of a 1D ultrathin planar semiconductor film (refractive index *n*_2_, extinction coefficient κ_2_, and thickness *d*_2_), replacing the 2D array of subwavelength elements in classical metasurfaces, and a transparent material spacer (thickness *d*_3_), sandwiched between the semi‐infinite transparent medium (1) and the metallic back reflector substrate. This structure is also representative of a solar cell configuration with medium (1) as the transparent electrode, the semiconductor film as the active layer, the transparent spacer as the carrier transport layer, and the metallic substrate as the metal electrode.

Here, we both theoretically and experimentally present a general strategy that facilitates the design of a planar metasurface comprising a 1D ultrathin planar semiconductor film (replacing the 2D array of subwavelength elements in classical metasurfaces), a transparent material spacer, and a metallic back reflector substrate as shown in **Figure** **1**. By using the simple building block, an ultrathin planar semiconductor film, we experimentally demonstrated a new type of macroscopic near‐perfect solar absorbers with nearly all the light absorbed in the semiconductor layer in contrast to other metamaterial and metasurface absorbers where light is mainly absorbed in their metallic components,^10^ missing the generation of electron–hole pairs for PV and PEC cells. Needless to say, the degree of complexity in fabricating 1D structures is orders of magnitude less compared to 2D and 3D structures. Although absorbers with similar planar structures (an ultrathin semiconductor layer coated on a metallic substrate with finite optical conductivity^13^ or with near zero permittivity,^14^ or on a polar dielectric substrate in its reststrahlen band^15^) have been studied, our design strategy is methodologically different. The strategy in refs. 13–15 is to use asymmetric Fabry–Perot optical cavities to excite the Gires–Tournois resonance, so these studies focused on designing the reflection phase shift at the interface between the semiconductor layer and the substrate^16^, ^17^ to achieve destructive interference yet with absorbed energy distribution less investigated. Our metasurface strategy is to tune not only reflection phase shift, that is, optical cavity behaviors, but also electromagnetic energy dissipation, that is, dissipative behaviors, to achieve near‐perfect absorption specifically in the semiconductor film, which is of crucial importance for solar PV and PEC applications.

To demonstrate the power and simplicity of this strategy, a prototype of planar metasurface solar cell was experimentally demonstrated and high‐efficiency colored planar metasurface perovskite solar cells were further designed. They hold great promise for energy‐efficient buildings because they can be integrated with both interiors and exteriors, such as facades, windows, and offices of the buildings. So far, two main approaches have been used to improve perovskite solar cell aesthetic. The first is to employ thick opaque perovskite layers (several hundreds of nanometers thick) with reflected colors generated from the nanophotonic structures incorporated on the top or at the bottom of the cells.^18^, ^19^, ^20^ Another is to use thin semitransparent perovskite layers (≈100 nm) with transmitted colors determined by the energy band structures of the perovskite layers^21^ or by the Fabry–Perot resonances supported in the electrodes.^22^, ^23^ However, these colored solar cells have shown low power conversion efficiency (below 9% in Zhang et al.^20^ and below 5% in Lee et al.,^22^ for example). Planar metasurface is used here as an alternative approach where the perovskite solar cell employs an ultrathin perovskite film (tens of nanometers in thickness) but have reflected color. The solar cell is designed to have a perovskite film that is one order of magnitude thinner than that in the regular black solar cell, but this cell was predicted to maintain 75% of the efficiency of its black counterpart. In addition, our designed solar cells display tunable and angle‐insensitive colors with purities comparable to those of state‐of‐the‐art color filters.^24^

## 2 Results and Discussion

### 2.1 Proof of Concept for Planar Metasurfaces

#### 2.1.1 Theoretical Design

As shown in Figure 1, eight parameters (*n*_1_, *n*_2_, κ_2_, *d*_2_, *n*_3_, *d*_3_, *n*_4_, and κ_4_) are involved in designing the planar metasurface absorber. What is lacking is a general theoretical framework to design this absorber in a more direct and quantitative way. The well‐known Gires–Tournois interferometers used in previous studies^13^, ^14^, ^15^ only design the reflection phase shift behavior, that is, the optical cavity behavior, but ignores the dissipative properties of the absorbers. In this section, we derived a new formalism to elucidate the absorption in the semiconductor film for the planar metasurface shown in Figure 1, which is very important for solar PV applications. No restriction was placed on the metallic substrate in this work, so even metals approaching the perfect electric conductor limit can be used; and this is in contrast to previous references^13^, ^14^ where metals with finite optical conductivity or near zero permittivity were used.

In the first step, we investigated the dissipative properties of planar metasurfaces. We have derived the expression to calculate the absorption rate per unit length (absorbed energy at a given depth) for multilayer structures based on the transfer matrix theory in our previous works.^16^, ^17^ Now we applied the theory to the ultrathin semiconductor film with the absorption rate per unit length normalized to the incident energy expressed as

(1) $$A_{\mathrm{pul}}\left(z,\lambda \right)=\frac{4\pi n_{2}\kappa_{2}}{\lambda n_{1}}\frac{{\left|E\left(z\right)\right|}^{2}}{{\left|E_{0}\right|}^{2}}$$

for normal incidence (assuming transverse‐electric polarized light), so the exclusive absorption in the ultrathin semiconductor film is

(2) $$A_{\mathrm{exc}}\left(\lambda \right)=\frac{4\pi n_{2}\kappa_{2}}{\lambda n_{1}}\frac{\int_{0}^{d_{2}}{\left|E\left(z\right)\right|}^{2}dz}{{\left|E_{0}\right|}^{2}}$$

where *E*(*z*) is the electric field in the ultrathin semiconductor film, *E*_0_ is the incident electric field, and λ is the wavelength. Zero reflection gives

(3) $$E\left(z=0\right)=E_{0}$$

Since the semiconductor film is much thinner than the wavelength and the electric field can penetrate into the transparent material spacer for *d*_3_ > 0, we assume that

(4) $$E\left(z\right)=E\left(z=0\right)=E_{0},\;0\leq z\leq d_{2}$$

This assumption is different from that in Park et al.^25^ and will be validated later. By substituting Equation (4) into Equation (2) and applying the perfect exclusive absorption condition (*A*_exc_ = 1), we get

(5) $$\frac{4\pi n_{2}\kappa_{2}d_{2}}{\lambda n_{1}}=1$$

In the next step, we studied the reflection phase shift behavior at the interface between the semiconductor film and the transparent spacer similar to previous studies.^13^, ^14^, ^15^, ^16^, ^17^, ^25^, ^26^, ^27^ The destructive interference condition, that is, the Gires–Tournois resonant condition, is

(6) $$\psi_{\mathrm{prop}}+\psi_{234}-\psi_{12}=\pi$$

where ψ_12_ is the reflection phase shift at the interface between medium (1) and the semiconductor film (1–2 interface), ψ_prop_ is the propagation phase shift in the semiconductor film, and ψ_234_ is the reflection phase shift at the interface between the semiconductor film and the transparent spacer (2–3 interface). ψ_12_ is equal to the phase angle of *r*_12_, the reflection coefficient at the 1–2 interface, expressed as

(7) $$r_{12}=\frac{m_{1}-m_{2}}{m_{1}+m_{2}}$$

where *m_p_* = *n_p_* + *iκ_p_* is the complex refractive index of layer *p*. ψ_234_ is equal to the phase angle of *r*_234_, the reflection coefficient at the 2–3 interface, expressed as

(8) $$r_{234}=\frac{r_{23}+r_{34}e^{2i\beta_{3}}}{1+r_{23}r_{34}e^{2i\beta_{3}}}$$

where *r_pq_* = (*m_p_* − *m_q_*)/(*m_p_* + *m_q_*) and β_3_ = 2*πn*_3_ *d*_3_/λ. ψ_prop_ is expressed as

(9) $$\psi_{\mathrm{prop}}=4\pi n_{2}d_{2}/\lambda$$

Two assumptions, (i) the magnitude of the complex refractive index of the semiconductor material is much larger than one which is reasonable for most semiconductors and (ii) the metallic substrate is approximated as perfect electric conductor, were applied to get

(10) $$\psi_{12}=\pi$$

(11) $$r_{34}=-1$$

(12) Imr234=Csin2β3

where Im(*r*_234_) is the imaginary component of *r*_234_ and *C* is a negative real number. Substituting Equations (9) and (10) into Equation (6) gives

(13) $$\frac{4\pi n_{2}d_{2}/\lambda +\psi_{234}-\pi }{\pi }=1$$

Equation (12) shows that ψ_234_ can always be designed to be larger than π, that is, Im(*r*_234_) < 0, by tuning the transparent spacer thickness, *d*_3_, so that we can find the semiconductor film thickness, *d*_2_, that is smaller than a quarter wavelength, circumventing the quarter‐wavelength lower limit, as well as satisfies Equation (13). The semiconductor film thickness, *d*_2_, can be designed by Equation (5) and then to accurately design the transparent spacer thickness, *d*_3_

(14) $$\frac{4\pi n_{2}d_{2}/\lambda +\psi_{234}-\psi_{12}}{\pi }=1$$

should be used; and thus, the planar metasurface absorber is designed.

Overall, by scrutinizing both the dissipative and optical cavity behaviors of the planar metasurface, we introduce Equations (5) and (14) as design guidelines to realize perfect absorption in the ultrathin semiconductor film at the target wavelength. We must point out that Equation (5) is valid for only the structure with semiconductor film much thinner than the wavelength, that is, in the ultrathin regime, so now we consider a limiting case where *d*_3_ = 0. This gives ψ_234_ = π. Substituting this result into Equation (13) gives *d*_2_ = λ/(4*n*_2_) corresponding to the quarter‐wavelength thickness limit. *d*_3_ = 0 also gives *E*(*z* = *d*_2_) = 0 because the electric field cannot penetrate into the perfect electric conductor substrate; the resultant $\int_{0}^{d_{2}}|E(z){|}^{2}dz$ is approximated to be equal to |*E*_0_|^2^/2. Perfect absorption leads to κ_2_ =*λn*_1_/(2*πn*_2_ *d*_2_). For the quarter‐wavelength thickness limit, we have κ_2_ = 2*n*_1_/π. Therefore, perfect absorption can be achieved in the ultrathin semiconductor film only for spectra where the extinction coefficient of the semiconductor material is larger than 2*n*_1_/π. Otherwise, although the planar metasurface absorber can resonate at the target wavelength determined by Equation (14), the absorption can never approach 100%.

#### 2.1.2 Experimental Demonstration

**Figure 2:**
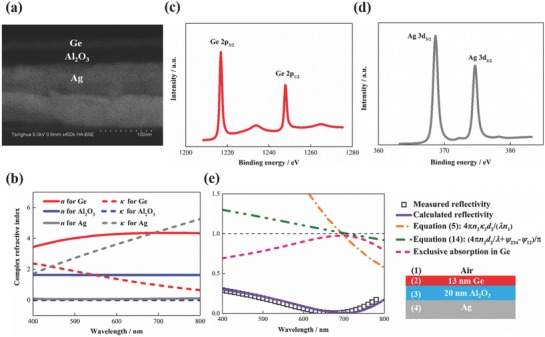
Demonstration of a macroscopic planar metasurface absorber. a) Schematic and backscattered electron image of the sample consisting of 13 nm Ge, 20 nm Al_2_O_3_, and 150 nm Ag coated on a silicon substrate fabricated using the electron beam evaporation method. b) Measured complex refractive indices of Ge, Al_2_O_3_, and Ag. c) Ge 2p spectra near the Ge–Al_2_O_3_ interface and d) Ag 3d spectra near the Al_2_O_3_–Ag interface. e) Measured and calculated reflectivity, and calculated exclusive absorption in Ge, 4*πn*_2_κ_2_ *d*_2_/(*λn*_1_) and (4*πn*_2_ *d*_2_/λ + ψ_234_ − ψ_12_)/π of the sample for normal incidence, demonstrating near‐perfect exclusive absorption in the ultrathin Ge layer.

Now we experimentally prove the concept. Without losing generality, germanium (Ge) was used for the semiconductor film with alumina (Al_2_O_3_) for the transparent spacer, air for medium (1) and silver (Ag) for the metallic substrate as shown in **Figure** **2**a; and the target resonant wavelength was selected to be 700 nm. The complex refractive indices of Ge, Al_2_O_3_, and Ag were measured using the ellipsometry method (see the Experimental Section) and shown in Figure 2b. The refractive index of Ge is 4.35 and the extinction coefficient is 0.96 at 700 nm wavelength. The Ge thickness was designed to be 13 nm, only 1/12 of the resonant wavelength, according to Equation (5) and the Al_2_O_3_ thickness was then designed to be 20 nm according to Equation (14). The 13 nm Ge/20 nm Al_2_O_3_/Ag sample was fabricated using the electron beam evaporation method, and characterized using the scanning electron microscopy shown in Figure 2a to show it is as designed. The Ge–Al_2_O_3_ and Al_2_O_3_–Ag interfaces were characterized using the X‐ray photoelectron spectroscopy shown in Figure 2c,d to show the sample is reliable though fabricated by a simple deposition method. We measured the spectral reflectivity of the sample using the spectrophotometer and calculated the spectra using the transfer matrix method (see theExperimental Section). Figure 2e shows that the calculated spectral reflectivity agrees well with experimental data; the reflectivity is below 1% and the exclusive absorption in Ge is higher than 97%, that is, near perfect absorption, at 700 nm wavelength. Results also show that the calculated 4*πn*_2_κ_2_ *d*_2_/(*λn*_1_) and (4*πn*_2_ *d*_2_/λ + ψ_234_ − ψ_12_)/π are both nearly equal to one corresponding well to Equations (5) and (14). Hence, they are effective design guidelines to maximize the exclusive absorption in the ultrathin semiconductor film for planar metasurfaces.

**Figure 3:**
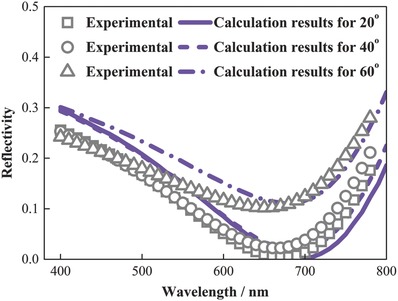
Measured and calculated reflection spectra of the planar metasurface sample for various incident angles up to 60°.

Furthermore, the planar metasurface exhibit omnidirectional resonant behaviors, which is also an important feature for solar PV applications. We measured and calculated the spectral reflectivity of the sample for unpolarized light with incident angles up to 60° as shown in **Figure** **3**. Results show that the calculated spectra are redshifted by less than 20 nm with respect to the measured spectra, so calculation results agree reasonably well with experimental data. The reason for this redshift is that the actual thickness of the deposited Ge film could be slightly smaller than 13 nm. Results also show that the resonant wavelength for unpolarized 60° incident light is blueshifted by only 20 nm from that for normal incident light. The reason for this angle robust behavior is as follows. The resonant wavelength is determined by Equation (6). Since the thicknesses of the Ge and Al_2_O_3_ layers are much smaller than the wavelength and Ge refractive index is as larger as 4.35, both the propagation and reflection phase shifts, and thus, the resonant wavelength, change slightly with incident angles. More detailed analysis can be referred to our previous work.^27^

### 2.2 Design of Solar Cells with Planar Metasurfaces

#### 2.2.1 Device Demonstration

**Figure 4:**
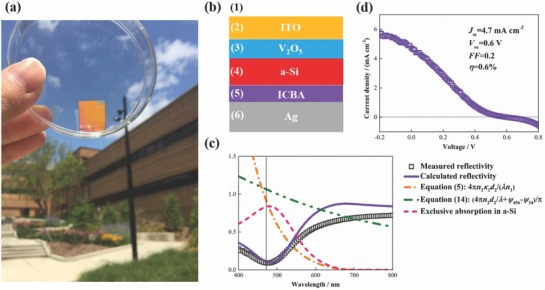
Demonstration of a prototype of planar metasurface solar cell. a) Image of the fabricated device with yellowish appearance. b) Device configuration. The solar cell consists of a 90 nm ITO‐coated glass substrate, an 8 nm thick V_2_O_5_ hole transport layer, a 14 nm thick a‐Si active layer, a 5 nm thick ICBA electron transport layer, and a 150 nm thick and opaque Ag cathode. c) Measured and calculated reflectivity, and calculated exclusive absorption in a‐Si, 4*πn*_4_κ_4_ *d*_4_/(*λn*_1_) and (4*πn*_4_ *d*_4_/λ + ψ_456_ − ψ_34_)/π of the sample for normal incidence. d) Current–density/voltage curve of the device.

The planar metasurface structure shown in Figure 1 is also representative of a solar cell configuration with medium (1) as the transparent electrode, the semiconductor film as the active layer, the transparent spacer as the carrier transport layer and the metallic substrate as the metal electrode. Therefore, to demonstrate the power and simplicity of the planar metasurface strategy, we designed solar cells with planar metasurface structures. The amorphous silicon (a‐Si) solar cell, shown in **Figure** **4**a, was designed and fabricated (see the Experimental Section) as a prototype taking advantage of our experience.^28^ Figure 4b shows the device configuration with the indium tin oxide (ITO)‐coated glass substrate used for the anode, vanadium oxide (V_2_O_5_) for the hole transport layer, a‐Si for the active layer, indene‐C_60_ bisadduct (ICBA) for the electron transport layer, and an opaque Ag film for the cathode.

Without losing generality, the resonant wavelength was selected to be 470 nm to produce yellowish appearance as shown in Figure 4a. Guided by our planar metasurface strategy, a‐Si thickness was designed to be 14 nm, that is, ultrathin, and ICBA thickness was designed to be 0 nm, but we used a very thin (5 nm) layer of ICBA to extract photogenerated electrons more efficiently. Figure 4c shows that the calculated reflectivity agrees well with experimental data. Results show that the total reflectivity is lower than 10%, that is, near‐perfect total absorption, and the exclusive absorption in a‐Si approaches 83% at the resonant wavelength. The exclusive absorption for this structure is lower compared to the aforementioned Ge absorber because both ITO and V_2_O_5_ can absorb light at the resonant wavelength. Results also show that the calculated 4*πn*_4_κ_4_ *d*_4_/(*λn*_1_) and (4*πn*_4_ *d*_4_/λ + ψ_456_ − ψ_34_)/π are both close to one corresponding well to our theoretical framework.

The electrical performance of the ultrathin a‐Si solar cell was charaterized by current density–voltage measurements as shown in Figure 4d. This device had a short‐circuit current of 4.7 mA cm^−2^, an open‐circuit voltage of 0.6 V, and a fill factor of 0.2, yielding an efficiency of 0.6%. This efficiency is reasonable for a prototype considering the state‐of‐the‐art 10.2% efficiency for a single‐junction cell using a 250 nm intrinsic a‐Si film,^29^, ^30^ which is over 18 times thicker than our device. We must also point out that the efficiency of our device can be enhanced, for example, by using LiF/Al cathode of which the lower work function aligns better with ICBA LUMO (lowest unoccupied molecular orbital) and a‐Si conduction energy level than Ag, or by adding additional Alq_3_ layer to reduce contact resistance, or by tuning layer thicknesses to optimize electrical properties.^28^ However, these works are out of the scope of this paper.

#### 2.2.2 Perovskite Solar Cells

**Figure 5:**
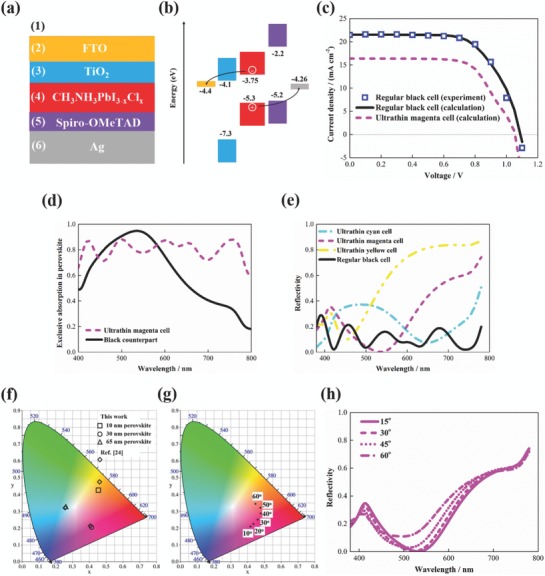
Performance and appearance of perovskite solar cells with planar metasurface structures. a) Device configuration. The solar cell consists of a CH_3_NH_3_PbI_3−_ *_x_*Cl*_x_* perovskite active layer, a 10 nm thick TiO_2_ electron transport layer, a 30 nm thick Spiro‐OMeTAD hole transport layer, a 100 nm thick FTO anode, and an opaque Ag cathode. 65, 30, and 10 nm thick perovskite films are used for ultrathin colored cells to generate cyan, magenta, and yellow colors, the standard CMY colors, with a 330 nm thick perovskite film for the regular black cell. TiO_2_, Spiro‐OMeTAD, and FTO thicknesses are on the same order of magnitude with those in previous studies. Although Spiro‐OMeTAD layers are usually several hundreds of nanometers thick to ensure high efficiency, tens of nanometers thick hole transport layers have been reported.^31^, ^32^ b) Band diagram. c) Current–density/voltage curves of the regular black cell (330 nm thick perovskite film) and the ultrathin magenta cell (30 nm thick perovskite film). Calculation results for the black cell agree well with the experimental data.^33^ The current–density/voltage curves of the ultrathin cyan and yellow cells are shown in Figure S1 (Supporting Information). d) Calculated exclusive absorption in perovskite of the ultrathin magenta and regular black cells for normal incidence. e) Calculated reflection spectra of the ultrathin colored cells and the benchmark black cell for normal incidence. f) Calculated color coordinates^34^ of the ultrathin cyan, magenta, and yellow cells for normal incidence. Calculated coordinates were compared to those of the color filters in ref. 24. g) Calculated color coordinates and h) reflectivity of the magenta cell for unpolarized light with various incident angles up to 60°. The reflection spectra of the cyan and the yellow cells for various incident angles are shown in Figure S1 (Supporting Information).

Perovskites have more suitable bandgaps, larger absorption coefficients, and longer carrier diffusion lengths^21^ compared to a‐Si and the state‐of‐the‐art efficiency of perovskite solar cells have exceeded 20%, twice of that of a‐Si solar cells.^29^ Therefore, we further designed colored perovskite solar cells that hold promise to meet all the desired characteristics which the ideal colored solar cell should have, including high power conversion efficiency, and high‐purity, tunable, and angle‐insensitive colors. This synergistic combination is achieved by using the ultrathin perovskite film to build planar metasurfaces. mixed halide perovskite (CH_3_NH_3_PbI_3−_ *_x_*Cl*_x_*) was used for the active layer as shown in **Figure** **5**a because Liu et al.^33^ reported that vapor‐deposited CH_3_NH_3_PbI_3−_ *_x_*Cl*_x_* films were more uniform with higher quality than solution‐processed films. TiO_2_ was used for the electron transport layer with Spiro‐OMeTAD for the hole transport layer, FTO for the anode, and Ag for the cathode, as used in the well‐known 15% efficiency cell.^33^ The energy band diagram of the perovskite solar cell is shown in Figure 5b.

It is of interest to compare the power conversion efficiency of the designed solar cell to its black counterpart by using our developed device model (see the Experimental Section). First, we benchmark the device model against the well‐known 15% efficiency black perovskite solar cell with a 330 nm thick perovskite film.^33^ The calculated current–density/voltage curve is shown in Figure 5c. The black cell has a short‐circuit current of 21.5 mA cm^−2^, an open‐circuit voltage of 1.09 V, a fill factor of 0.65, yielding power conversion efficiency of 15.3% as summarized in **Table** **1**. These calculated results were compared with the experimental data^33^ and they agree well as shown in Figure 5c and Table 1, verifying the accuracy of our device model. Then we reduce the perovskite film thickness by one order of magnitude. Without losing generality, the resonant wavelength was selected to be 535 nm to produce the magenta color. The perovskite film thickness was designed to be 30 nm with 30 nm thickness for the Spiro‐OMeTAD layer guided by our planar metasurface strategy. Figure 5d shows that the exclusive absorption in perovskite approaches 95%, that is, near‐perfect absorption, at 535 nm wavelength.

**Table 1:** Solar cell short‐circuit currents, *J*_sc_, open‐circuit voltages, *V*_oc_, fill factors, FF, and power conversion efficiency, η

	*J*_sc_ [mA cm^−2^]	*V*_oc_ [V]	FF	η [%]
Regular black cell (experiment)	21.5	1.07	0.67	15.4
Regular black cell (calculation)	21.5	1.09	0.65	15.3
Ultrathin magenta cell (calculation)a)	16.4	1.05	0.66	11.5

^a)^ Parameters of the ultrathin cyan and yellow cells are listed in Table S1 (Supporting Information).

The designed ultrathin magenta cell achieves a remarkable short‐circuit current of 16.4 mA cm^−2^, 76% of the photocurrent of its black counterpart (21.5 mA cm^−2^), despite the use of a perovskite film that is one order of magnitude thinner. This high photocurrent arises from the enhanced exclusive absorption over the resonant wavelength range which can partially compensate for the lower absorption in the rest of the visible spectrum compared to the black cell. As shown in Figure 5d, the magenta cell has higher exclusive absorption in perovskite for wavelengths from 445 to 580 nm. Results also show that the open‐circuit voltage decreases by 4% to 1.05 V after reducing the perovskite thickness to 30 nm. The solar cell with thinner semiconductor active layer has higher interfacial defect level which causes reduced open‐circuit voltage.^28^, ^35^ The fill factor of this cell increases by 2% to 0.66 because the thinner cell has less bulk recombination.^28^ The open‐circuit voltage and fill factor variations with perovskite thicknesses reported in this work and in refs. 21,28 follow the same trend. Overall, the ultrathin magenta cell achieves notable efficiency of 11.5%, maintaining 75% of the efficiency of its black counterpart. Although this efficiency is not experimentally demonstrated, it is feasible given the accurate device model and the mature fabrication technique.

Figure 5e shows that the benchmark cell has low reflection (≈10%) over the entire visible spectrum corresponding to a black appearance. On the contrary, the ultrathin magenta cell has low reflection (below 10%) from 475 to 580 nm while it is highly reflective with its optical properties dominated by the Ag cathode over the rest of the visible spectrum, especially for longer wavelengths; and thus, it is vividly colored with high purity. In addition, the perovskite thicknesses can be used to tune solar cell colors. 65, 30, and 10 nm thick perovskite films are designed to produce cyan, magenta, and yellow colors, the standard CMY colors, as shown in Figure 5f, with the corresponding spectral reflection dip at 650, 535, and 450 nm as shown in Figure 5e. By combining solar cells with various colors, arbitrary patterns or images can be obtained.

To demonstrate high‐purity and angle insensitivity features, we compared our designed solar cells to the state‐of‐the‐art angle robust color filters with planar structures.^24^ Calculated color coordinates in Figure 5f show that our cells and referenced filters have comparable color purities. Color coordinates of the magenta cell for various incident angles (viewing angles) were also calculated and Figure 5g shows that the cell color is nearly unchanged with viewing angles up to 60°, corresponding well to the reflection spectra in Figure 5h. Results show that the resonant wavelength for unpolarized 60° incident light is blueshifted by 40 nm from that for normal incident light, which is also comparable to the results in ref. 24.

## 3 Conclusion

In summary, a general strategy was both theoretically and experimentally presented to create a new type of macroscopic planar metasurface absorber consisting of a 1D ultrathin planar semiconductor film (instead of the 2D array of subwavelength resonators in classical metasurfaces), a transparent material spacer, and a metallic back reflector. Rather than following the well‐known nano‐optical‐cavity framework, we derived a new formulism to tune not only the optical cavity behaviors but also the dissipative properties to achieve near‐perfect absorption exclusively in the semiconductor film. To demonstrate the power and simplicity of this strategy, a protoype of planar metasurface solar cell was experimentally demonstrated. We further designed high‐efficiency colored perovskite solar cells with planar metasurface structures. The designed solar cells employ ultrathin perovskite films but display reflected colors. Our device model predicts that they can maintain 75% of the efficiency of their black counterparts despite the use of perovskite films that are one order of magnitude thinner. They can also display high‐purity (comparable to those of state‐of‐the‐art color filters) and angle‐insensitive (for viewing angles up to 60°) colors. This work paves the way to miniaturized, planar, and multifunctional solar cells and optoelectronic devices.

## 4 Experimental Section

*Experiment*: Electron beam evaporation was used to deposit the multilayer structure shown in Figure 2a, and to deposit thin films for complex refractive index measurements as shown in Figure 2b. Ge, Al_2_O_3_, and Ag were deposited for rates of 0.1, 0.1, and 0.3 nm s^−1^ and pressures of ≈10^−6^ torr. Their complex refractive indices were measured using the ellipsometry method. The multilayer structure sample was characterized using scanning electron microscopy and X‐ray photoelectron spectroscopy as shown in Figure 2a,c,d. The reflectivity spectra in Figures 2e and 3 were measured using the PerkinElmer Lambda 950 spectrophotometer combined with the universal reflectance accessory. The expanded uncertainty is 0.5% for near‐normal, 20° and 40° incidence, and 2% for 60° incidence. Regarding the prototype device, V_2_O_5_ and Ag were deposited for rates of 0.02 and 0.1 nm s^−1^ and pressures of ≈10^−6^ torr by thermal evaporation. ICBA was spin cast. The a‐Si was deposited using the plasma‐enhanced chemical vapor deposition method. The device was tested for AM1.5 simulated sunlight illumination condition. The instrument of Keithley 2400 was used for the data acquisition of the current and voltage.

*Theory*: The expression of the absorption rate per unit length normalized to the incident energy was derived based on the transfer matrix theory. This theory was also used to calculate the reflectivity and the exclusive absorption in the semiconductor film. The device model features coupled electrical and optical modeling. The transport of photogenerated charge carriers is governed by the diffusion and the electrically induced drift equations. The electrostatic potential and carrier concentrations throughout the device were calculated by solving the Poisson and the continuity equations. The photogeneration rate was calculated using the transfer matrix method. The generated charge carriers can recombine via radiative, Auger, Shockley–Read–Hall, and surface mechanisms. The optical parameters from refs. 36,37 and the electrical parameters from refs. 38,39 were used for modeling. The surface recombination velocities for ultrathin colored cells were assumed to be one order of magnitude larger than that for the regular black cell, because the cells with thinner semiconductor active layers have higher interfacial defect level.^28^, ^35^

## Conflict of Interest

The authors declare no conflict of interest.

## Supporting information

Supplementary
