# Supplementary material for: Planar Metasurfaces Enable High‐Efficiency Colored Perovskite Solar Cells
Source: Adv Sci (Weinh). 2018 Aug 26;5(10):1800836. doi: 10.1002/advs.201800836 (PMC6193155; doi:10.1002/advs.201800836)
Supplement: Supplementary file 1 — Supplementary [file ADVS-5-1800836-s001.pdf]

Copyright WILEY-VCH Verlag GmbH & Co. KGaA, 69469 Weinheim, Germany, 2018.

ADVANCED SCIENCE
Open Access

Supporting Information

for *Adv. Sci.*, DOI: 10.1002/advs.201800836

Planar Metasurfaces Enable High-Efficiency Colored Perovskite Solar Cells

Dong Liu, Lin Wang, Qingyu Cui, and L. Jay Guo*

WILEY-VCH

Copyright WILEY-VCH Verlag GmbH & Co. KGaA, 69469 Weinheim, Germany, 2016.

Supporting Information

Title: Planar Meta-Surfaces Enable High-Efficiency Colored Perovskite Solar Cells

Dong Liu, Lin Wang, Qingyu Cui, and L. Jay Guo*

Prof. D. Liu, L. Wang
MIIT Key Laboratory of Thermal Control of Electronic Equipment, School of Energy and Power Engineering, Nanjing University of Science and Technology, Nanjing 210094, China.
E-mail: liudong15@njust.edu.cn
Q. Cui, Prof. L. J. Guo
Department of Electrical Engineering and Computer Science, University of Michigan, Ann Arbor, Michigan 48109, USA

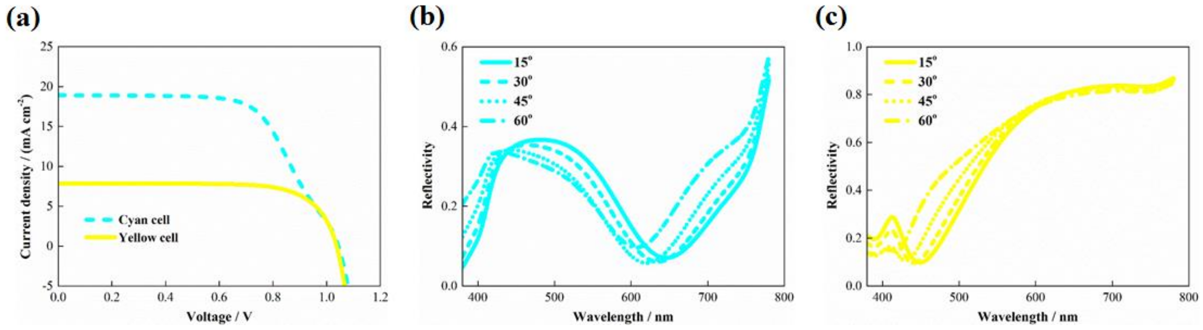

Figure S1. (a) Current-density/voltage curves of ultrathin cyan and yellow cells. Calculated reflection spectra of the (b) cyan and (c) yellow cells for unpolarized light with various incident angles up to 60°.

Table S1. Solar cell short-circuit currents, J_{sc}, open-circuit voltages, V_{oc}, fill factors, *FF*, and power conversion efficiency, η.

	J_{sc} (mA cm^{-2})	V_{oc} (V)	*FF*	η (%)
Cyan cell	18.9	1.05	0.63	12.6
Magenta cell	16.4	1.05	0.66	11.5
Yellow cell	7.8	1.04	0.73	6.0
